# Dynamic Network Analysis Reveals Altered Temporal Variability in Brain Regions after Stroke: A Longitudinal Resting-State fMRI Study

**DOI:** 10.1155/2018/9394156

**Published:** 2018-04-05

**Authors:** Jianping Hu, Juan Du, Qiang Xu, Fang Yang, Fanyong Zeng, Yifei Weng, Xi-jian Dai, Rongfeng Qi, Xiaoxue Liu, Guangming Lu, Zhiqiang Zhang

**Affiliations:** ^1^Department of Radiology, The First Affiliated Hospital of Fujian Medical University, Fuzhou, China; ^2^Department of Medical Imaging, Jinling Hospital, Nanjing Clinical School, Southern Medical University, Nanjing, China; ^3^Department of Neurology, Jinling Hospital, Medical School of Nanjing University, Nanjing, China; ^4^Department of Medical Imaging, Jinling Hospital, Medical School of Nanjing University, China; ^5^State Key Laboratory of Analytical Chemistry for Life Science, Nanjing University, Nanjing, China

## Abstract

Recent fMRI studies have demonstrated that resting-state functional connectivity (FC) is of nonstationarity. Temporal variability of FC reflects the dynamic nature of brain activity. Exploring temporal variability of FC offers a new approach to investigate reorganization and integration of brain networks after stroke. Here, we examined longitudinal alterations of FC temporal variability in brain networks after stroke. Nineteen stroke patients underwent resting fMRI scans across the acute stage (within-one-week after stroke), subacute stage (within-two-weeks after stroke), and early chronic stage (3-4 months after stroke). Nineteen age- and sex-matched healthy individuals were enrolled. Compared with the controls, stroke patients exhibited reduced regional temporal variability during the acute stages, which was recovered at the following two stages. Compared with the acute stage, the subacute stage exhibited increased temporal variability in the primary motor, auditory, and visual cortices. Across the three stages, the temporal variability in the ipsilesional precentral gyrus (PreCG) was increased first and then reduced. Increased temporal variability in the ipsilesional PreCG from the acute stage to the subacute stage was correlated with motor recovery from the acute stage to the early chronic stage. Our results demonstrated that temporal variability of brain network might be a potential tool for evaluating and predicting motor recovery after stroke.

## 1. Introduction

Functional connectivity (FC) and functional connectomics based on resting-state functional magnetic resonance imaging (rs-fMRI) have proven to be powerful tools for investigating the brain function in both physiological and disease states [1–3]. Most of the studies on resting-state brain networks were based upon the assumption that the strength of FC is constant over scan session [4]. Recently, merging evidence suggests that the FC of the resting-state brain network is not static and presents temporal variability even within a session [5–7]. Temporal variation in FC, the so-called dynamic functional connectivity, can be attributed to neural activity [7, 8]. Furthermore, disease-related alterations in the dynamic properties of FC have also been reported, suggesting that temporal features of FC could serve as a disease biomarker [9, 10].

Recently, a novel approach introduced by Zhang et al. could measure the temporal variability of functional architecture in a specific region, which is different from the conventional dynamic FC method which only measures the interregional property of FC variability [11]. The temporal variability of the particular brain region might reflect its dynamic reconfiguration into distinct functional regions within the brain network at different times and might be an indicator of brain flexibility and adaptability [11]. Therefore, the temporal variability may provide new insight into the pathophysiological mechanisms of neural rehabilitation after brain damage such as the neural process of recovery after stroke.

Motor function impairment is one of the most common complications of stroke. Following initial cerebral damage, motor function in nearly all stroke patients can usually recover to some extent within the first six months after stroke [12]. However, the underlying mechanism of motor recovery after stroke is not entirely understood. A large number of fMRI studies have shown that motor recovery after stroke is a complicated process related to the cerebral structural and functional reorganization [13–17]. However, most of these studies have been confined to the exploration of static functional connectivity or networks, and few studies have carried out the approach of temporal variability.

In the present study, a longitudinal data of stroke patients with motor impairment across three consecutive stages was used to investigate the dynamic topological properties of brain networks by measuring the temporal variability of whole-brain functional networks. The first stage referred to as the acute stage was within the first week after symptom onset. The second stage referred to as the subacute stage was between one week and two weeks after the stroke onset. The third stage defined as the early chronic stage was at 3-4 months after the stroke onset [15]. Besides, we also introduced a group of age-matched healthy controls as the baseline. We sought to determine (1) how the temporal variability of brain functional network changes over the course of motor recovery after stroke and (2) whether the alterations of the regional temporal variability of brain functional network are associated with motor recovery. Exploring these issues could contribute to understanding the neurophysiological mechanisms for motor rehabilitation after stroke.

## 2. Materials and Methods

### 2.1. Participants

The local Ethical Committee approved the experiment, and all subjects signed the informed consent before the study. Nineteen patients (17 male, mean age 52.26 ± 11.73 years, 12 left-side deficits) with acute first-ever ischemic stroke were enrolled from January 2015 to March 2016. The inclusion criteria were as follows: (1) first-ever ischemic stroke, (2) unilateral hand motor deficit, (3) symptom onset < 7 days, (4) age between 18 and 80 years, and (5) single stroke lesion located within the middle cerebral artery territory on MRI. The exclusion criteria consist of (1) hemorrhagic stroke, (2) bilateral stroke lesions on MRI, (3) language or cognitive deficits sufficient to affect informed consent, (4) other orthopedic, neurological, or psychiatric diseases substantially affecting the arm, (5) contraindications to MRI examination, and (6) recurred stroke during the follow-up. In addition, 19 healthy right-handed participants (12 men; 7 women; mean age 51.1 ± 5.99 years) were enrolled as the baseline.

Clinical measures and neuroimaging data were assessed at three consecutive time points during the poststroke phase. The mean time intervals between stroke onset and MRI scan points were as follows: the acute stage (4.11 ± 1.76 days poststroke, ranging 1–7 days), the subacute stage (10.47 ± 2.12 days poststroke, ranging 8–14 days), and the early chronic stage (99.32 ± 8.54 days poststroke, ranging 87–116 days). The lesion volumes were calculated at the acute stage. The mean lesion size was 3. 26 ± 1. 93 ml (ranging 0.61–6.44 ml). The lesion map of each patient on the diffusion-weighted image was shown in Supplementary Figure 1. The hand motor function of each patient was assessed using an upper limb Fugl-Meyer Motor Assessment (UL-FMA), which has a range of 0 (complete hemiplegia) to 66 (normal performance) for upper extremities. The mean UL-FMA scores at three stages were as follows: 33.58 ± 14.55 (ranging 6–56) in the acute stage, 42.63 ± 16.79 (ranging 7–61) in the subacute stage, and 55.11 ± 12.27 (ranging 27–66) in the early chronic stage. Demographic and clinical characteristics of stroke patients were shown in Table 1. The specific information of each patient was shown in Supplementary Table 1.

### 2.2. MRI Image Data Acquisition

All MR images were acquired using a 3.0T whole-body scanner (Discovery MR750, GE Healthcare, Milwaukee, WI, USA) with a 32-channel phased-array head coil. The participants were placed on the scanner gantry in a head-first supine position with a plastic holder to minimize head motion and earplugs to reduce scanner noise. High-resolution 3D T1-weighted structural images were obtained in the transverse orientation using a 3D-BRAVO sequence with the following parameters: TR = 8.2 ms, TE = 3.2 ms, flip angle = 12°, FOV = 220mm × 220mm, matrix = 256 × 256, and slice thickness = 1.0 mm. Functional MR images were acquired with a gradient-echo EPI sequence with the following scan parameters: TR = 2000 ms, TE = 30 ms, flip angle = 80°, FOV = 240mm × 240mm, matrix = 64 × 64, slice thickness = 3.0 mm, no gap, and slice number = 43. The scan range covered the whole brain tissue from the vertex to the lower parts of the cerebellum. During the rs-fMRI scans, patients were instructed to keep their eyes closed, think of nothing in particular, and not fall asleep. Scan time lasted 6 minutes 50 second, and a total of 205 volumes were acquired.

### 2.3. fMRI Data Preprocessing

**Figure 1:**
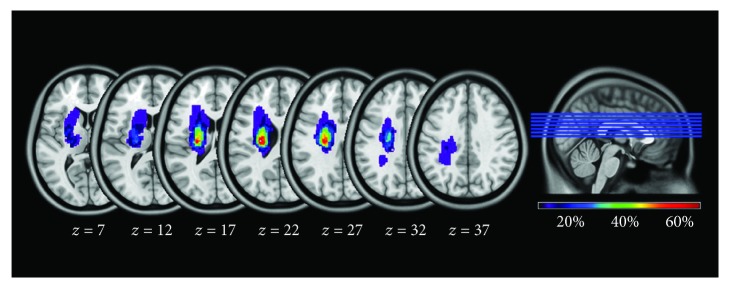
The overlap map of the lesions across all the stroke patients during the acute stage. Color bar indicates the percentage of the lesion overlap. *z*-axis from 7 to 37 in MNI coordinates. MNI: Montreal Neurological Institute.

fMRI data preprocessing was conducted by SPM8 (http://www.fil.ion.ucl.ac.uk/spm) and Data Processing Assistant for Resting-State fMRI (DPARSF) [18]. Before data preprocessing, the imaging data of patients with right hemisphere lesions were flipped from the right to the left along the midsagittal plane. Thus, after flipping, the left hemisphere corresponded to the ipsilesional side and the right hemisphere corresponded to the contralesional side in all patients. The lesion over map across all patients in the acute stage was shown in Figure 1.

### 2.4. Process of Structural MRI Data

To avoid tissue misclassification led by infarcted tissue during segmentation and normalization, we used a cost function method to remove the influence of lesions [19, 20]. The specific procedures were followed: firstly, the lesion mask was created on the individual 3D T1-weighted structural images by two radiologists (Hu JP and Zeng FY), with the guide of a DWI image. Secondly, a group-sample-specific brain template was generated. For each subject, the lesion-removed whole-brain mask was used as the cost function to normalize the 3D T1-weighted structural image into the standard brain template in MNI (Montreal Neurological Institute) space by using a 12-parameter affine transformation with nonlinear adjustments with 7 × 8 × 7 basis functions. All individually normalized 3D T1-weighted structural images and all lesion-removed masks were averaged to yield a sample-specific brain template. Thirdly, the averaged 3D T1-weighted structural template was segmented using the unified segment function of SPM8, with the averaged lesion-removed mask as the cost-function. Fourth, the individual space 3D T1-weighted structural images were segmented using unified segment function. The segment issues in the third step process were used as the templates for the current segment process, and the individual lesion-removed brain mask was used as the cost function. Finally, the segment parameters containing the affine transition matrix were generated, which would be used in the functional MRI image normalization.

### 2.5. Analysis of Resting-State Functional Image Data

Firstly, the first five volumes of each run were discarded to allow for the signal to reach equilibrium and for the patients to adapt to the scanning noise. The remaining 200 volumes were corrected for slice timing effects for each volume, and then all volumes were realigned to the first volume to adjust for the residual head movement. The estimated translation or rotation parameters did not exceed 1.5 mm or 1.5 degrees. Secondly, the realigned images were coregistered to the individual 3D T1-weighted images. Thirdly, the segment parameters gained in structural MRI data process were applied to the coregistered images for the normalization of functional images, and then the functional images were resampled to 3 × 3 × 3 mm^3^ voxel size. After normalization, the BOLD signal of each voxel in resting-state functional MR images was first detrended to abandon linear trend and then temporal band pass filter (0.01–0.08 Hz) was performed to reduce low-frequency drift and high-frequency physiological noise. Finally, sources of spurious variance, including head motion parameters and white matter, cerebrospinal, and global signals, were removed through linear regression.

### 2.6. Brain Regional Temporal Variability Analysis

**Figure 2:**
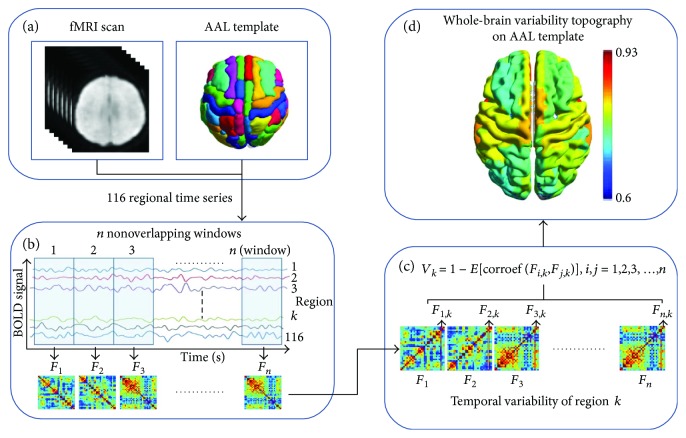
Definition of temporal variability for a given region *k* in the functional network. (a) The BOLD time series for 116 brain regions were extracted from AAL template. (b) All BOLD time series were segmented into *n* nonoverlapping windows. Functional networks are constructed in each time window. (c) The temporal variability of the region *k* is determined by comparing functional connectivity profile of the region *k* at different windows. (d) The topographic pattern of temporal variability in the whole brain based on the AAL template was demonstrated.

The AAL (Automated Anatomical Labeling) template was used to parallel the brain into 116 ROIs (regions of interest). The time series of ROIs were gained by averaging the time series of all voxels in the corresponding ROIs [21, 22], and 116 regional time series were obtained for the later analysis. According to the steps described by Zhang et al. [11], the temporal variability of each brain region was calculated. The specific steps were as follows (Figure 2): firstly, all BOLD time series were segmented into *n* nonoverlapping windows each with a window length *l*. Within the *i*th time window, the whole brain functional connectivity network *F*_*i*_ (a 116 × 116 matrix, with 116 brain regions) was obtained using Pearson correlation analysis. Secondly, the functional architecture of a region *k* at the *i*th time window was defined as the overall functional connectivity profile of region *k*, which was a 116-dimensional vector and was shortened as *F*_*i*,*k*_. Thirdly, the temporal variability of a region of interest *k* was computed by comparing the functional connectivity profile of region *k* at different windows. The concrete formulation was listed as follows:

(1) $$\begin{array}{c} V_{k}=1-\overset{¯}{corrcoef\left(F_{i,k},F_{j,k}\right)}, \end{array}$$

where *i*, *j* = 1, 2, 3,…, *n*, *i* ≠ *j*.

Here, *i*, *j* meant different time windows and *n* meant the number of total windows determined by window length *l*. Finally, to avoid the arbitrary choice of window length in the study, we calculated *V*_*k*_ at different window lengths *l* (*l* = 10, 11, 12,…, 20 volumes). The average value of all *V*_*k*_ values over different window lengths was defined as the final temporal variability of the ROI.

### 2.7. Statistical Analysis

Statistical tests were performed using the MATLAB 2016a statistical package and SPSS version 22 (Statistical Package for the Social Sciences, IBM). Results are expressed as mean ± standard deviation (SD). Two-sample *t*-test was used to investigate the significant difference in regional temporal variability of brain networks between the stroke patients and healthy controls. One-way repeated measures ANOVA and multiple comparisons (post hoc Tukey's test) was used to assess the significant differences in UL-FMA score and temporal variability in brain regions between the three stages of stroke progression.

To investigate the link between temporal variability in brain regions and motor performance over the course of motor recovery, Pearson correlation analysis was used to analyze the correlation between the change in UL-FMA scores and the changes in temporal variability in brain regions with the significant difference between the three stages.

## 3. Results

### 3.1. Behavioral Data

One-way repeated measures ANOVA showed that the UL-FMA scores significantly increased over the process of motor recovery (*F* = 81.86, *P* < 0.001), and post hoc test further indicated that there was a significant increase in UL-FMA scores between the three stages (*P* < 0.001) following motor recovery.

### 3.2. Differences in Temporal Variability of Brain Networks between Healthy Controls and Stroke Patients

The difference in temporal variability of brain networks was evaluated between healthy controls and stroke patients during three stages (*P* < 0.005 with uncorrected, two sample *t*-test).

**Figure 3:**
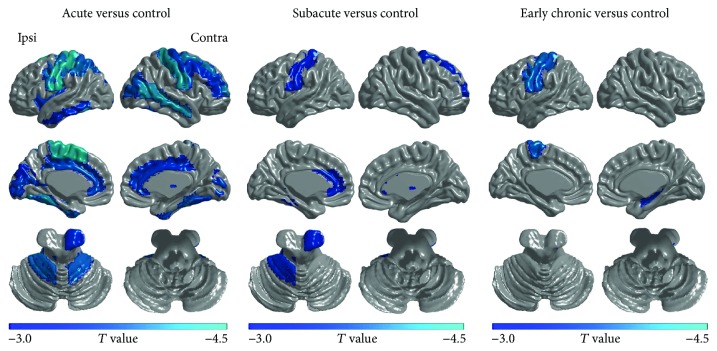
Brain regions showing significant differences in temporal variability between controls and patients during three stages (two-sample *t*-test, *P* < 0.005). All brain regions were summarized in Table 2. Ipsi: the ipsilesional side; Contra: the contralesional side.

Compared with the healthy controls, the stroke patients exhibited reduced temporal variability in all brain regions showing significant changes during the three stages which were summarized in Table 2 and Figure 3. The reduced temporal variability in brain regions covered the primary sensorimotor, auditory and visual cortices, and default mode network (DMN) which were found at the acute stage of the stroke group relative to healthy controls. Compared with the healthy controls, the stroke patients showed reduced temporal variability in ipsilesional postcentral gyrus (PoCG), ipsilesional anterior cingulate gyri (ACG), ipsilesional cerebelum_4_5, contralesional superior parietal gyrus (SFG), and contralesional thalamus at the subacute stage. Reduced temporal variability in the ipsilesional precentral gyrus (PreCG), ipsilesional PoCG, and contralesional hippocampus was found at the early chronic stage of stroke patients relative to healthy controls.

### 3.3. Longitudinal Changes in Temporal Variability of Brain Networks over the Stages of Stroke

**Figure 4:**
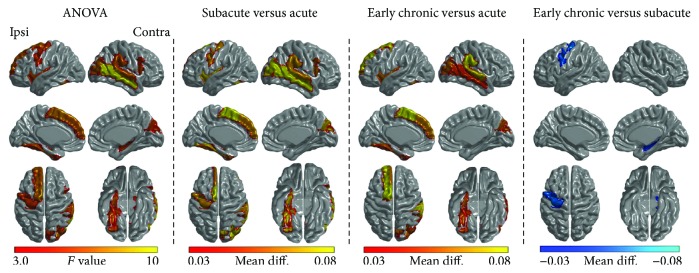
Brain regions showing significant differences in temporal variability across the three stages (one-way repeated measures ANOVA and post hoc test, *P* < 0.01 and *P* < 0.05, resp.). All brain regions were summarized in Table 3. Ipsi: the ipsilesional side; Contra: the contralesional side.

One-way repeated measures ANOVA showed that thirteen regions demonstrated significant differences in temporal variability among the three stages (*P* < 0.01, Table 3, Figure 4). Multiple comparisons were further performed to estimate the differences in these brain regions (Table 3, *P* < 0.05). Compared to the acute stage, the subacute stage showed increased temporal variability in several brain regions such as the primary motor, auditory, and visual cortices and most of these brain regions kept increasing temporal variability at the early chronic stage. Compared to the subacute stage, the early chronic stage showed reduced temporal variability in the ipsilesional PreCG and the contralesional hippocampus while the other eleven brain regions demonstrated increased temporal variability.

### 3.4. The Relationships between Temporal Variability in Brain Regions and Motor Function

**Figure 5:**
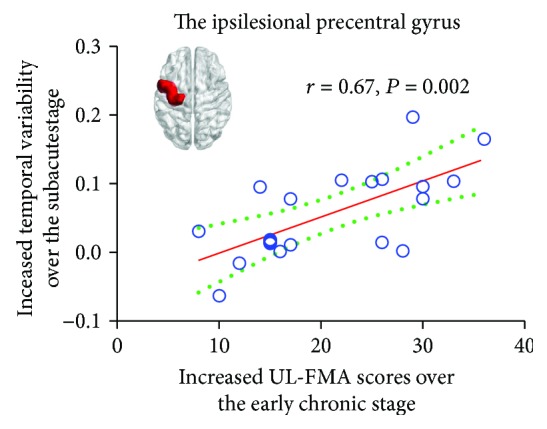
Correlation result between altered temporal variability and motor recovery (Pearson correlation analysis, *P* < 0.05). The increased temporal variability of the ipsilesional precentral gyrus over the subacute stage was positively correlated to the increased UL-FMA over the early chronic stage.

Motor recovery over the early chronic stage (increased UL-FMA from the acute stage to early chronic stage) significantly and positively correlated to change in temporal variability over the subacute stage (increased regional temporal variability from acute stage to subacute stage) in the ipsilesional PreCG (*r* = 0.67, *P* = 0.002, Figure 5). Moreover, there was also no significant correlation between motor recovery and change in temporal variability in other brain regions over any other session interval. This finding indicates that long-term motor recovery is related to change in temporal variability in the ipsilesional PreCG over the subacute stage.

## 4. Discussion

In the present study, we investigated the longitudinal alteration in temporal variability of resting-state functional networks in stroke patients with motor function impairment. Our findings showed that (1) compared with healthy controls, stroke patients at the acute stage demonstrated extensively reduced temporal variability in several brain regions such as primary sensorimotor, auditory, visual cortices, and DMN. The temporal variability in these brain regions was restored to normal level from acute the stage to the early chronic stage except the ipsilesional sensorimotor cortex and contralesional hippocampus. (2) We observed a time-dependent alteration in temporal variability of brain networks in the longitudinal study. Specifically, from the acute stage to the early chronic stage, the temporal variability in the ipsilesional PreCG and the contralesional hippocampus was increased first and then decreased, while the temporal variability of the other regions showed a trend of gradual increase. These findings suggest that the changes in the temporal variability may reflect dynamic reconfiguration of brain networks after stroke and may provide useful and complementary information for motor rehabilitation after stroke.

The temporal variability of a given brain region is considered to be negatively correlated to the variance of regional BOLD signal and energy of low-frequency component and to be positively correlated to the alpha-band oscillation-recorded electroencephalography [8]. Many studies have demonstrated that the slow-5 oscillations are particularly susceptible to disruption induced by the stroke. These studies showed that the stroke group exhibited an increasing trend in the low-frequency fluctuations at the acute stage (<7 days after stroke onset) and a decreasing trend from the acute stage to the early chronic stage, which may have been due to an inhibitory deficit from impaired the “task-positive” regions [23–25]. Besides, a decrease trend in alpha rhythm both in acute and in subacute stroke patients has been reported in EEG studies [26–28]. In our results, when compared to healthy controls, stroke patients exhibited reduced temporal variability in all brain regions showing significant changes during the acute stage. On the basis of these previous studies, we discreetly speculate that the disruption of function network integrity and the damage of regional neural activity induced by stroke could be responsible for the reduced temporal variability in brain regions after stroke, especially at the acute stage. Moreover, previous studies have indicated that the increase in BOLD signal variability is beneficial to more efficiently process and respond to unexpected external events [29, 30], and brain regions with more low-frequency components tend to be more easy to synchronize with other regions [11, 31]. At such condition, keeping low temporal variability in functional networks is also beneficial to maintain information transmission and functional integration with other brain regions.

In the longitudinal study, we found that temporal variability in brain regions of multiple functional networks, including DMN, attention network, and visual network, significantly increased and restored to normal level when compared with the healthy controls during the subacute stages. In line with these findings, several studies have pointed out that stroke recovery might involve the alternations of resting-state networks (RSNs), involving inter-RSNs and intra-RSNs [4, 32–34]. The temporal variability associated with a brain region might reflect its dynamical reconfiguration into distinct brain regions within the brain network [8]. Such an increase in temporal variability during the subacute stage may well be validated and explicated by many studies on stroke, in which excessive neuronal clustering and wiring were observed in the peri-infarct regions at the initial recovery phase. For example, previous studies have demonstrated that new structural circuits in both the perilesional zone and other distant regions would reestablish to compensate for the loss of function in the damaged cortex during the process of functional reorganization after stroke. What is more is that branch-specific remodeling of the dendritic arbor in the peri-infarct cortex dramatically increased within the first two weeks after stroke and is still evident six weeks after stroke [35, 36]. A brain network study based on animals also found that at the initial phases of poststroke recovery, neural spine elongation, neurite sprouting, might create a state of over connectivity that leads to the loss of signal synchronization and increase in random integration between neurons, resulting in reduced cortical signal coherence and FC [37]. Collectively, our findings suggest that rapidly increasing temporal variability in the related brain regions is an important feature in the reorganization and integration of resting-state functional network during the subacute stage.

Our results also demonstrated reduced temporal variability in the ipsilesional PreCG and the contralesional hippocampus during the early chronic stage. Reduced temporal variability within a given region means that communication between this region and the other regions remains in a high degree of synchronization across different time windows, which is consistent with increased FC between this region and other regions. These findings were in line with previous studies, in which the FC strength restoration of the ipsilesional PreCG with other brain regions was an essential feature during motor recovery after stroke [38]. Remarkably, the contralesional hippocampus, which usually exhibits high temporal variability in healthy subjects [11], showed reduced temporal variability during the early chronic stage. Although the underlying mechanism remains unclear, functional and structural alterations in the cognitive-related brain regions such as the hippocampus have been extensively reported to facilitate recovery of motor function in stroke patients [39–41]. In particular, reduced temporal variability in the contralesional hippocampus may be related to its increased gray matter volume [39].

A number of fMRI studies have demonstrated that early fMRI brain activation patterns were associated with subsequent motor recovery independent of initial motor impairment [42–45]. In the present study, we observed that increased temporal variability of the ipsilesional precentral gyrus over the subacute stage was positively correlated with the long-term motor recovery. Previous studies have pointed out that the reinstatement of previously reduced neural activity in the ipsilesional primary motor areas was the important feature of motor recovery after stroke [16, 42, 46–48]. Our result further strengthened the understanding for the important role played by the ipsilesional primary motor cortex in motor recovery after stroke and also indicated that altered temporal variability of the ipsilesional precentral gyrus might serve as a prediction indicator in motor recovery after stroke.

There are some limitations in the present study. Firstly, although we restricted the participants to stroke patients with single stroke lesion located within the middle cerebral artery territory, the location and size of stroke lesion exhibited a relative heterogeneity across the patients, which could create challenges in result interpretation. Secondly, although we adopted a longitudinal study design, three months follow-up with three time points is relatively short. A longer follow-up period with more time points is helpful to fully understand the relationship between temporal variability of functional network and stroke recovery in the future study. Finally, the impact of flipping image data on results is unclear.

In summary, using the dynamic network analysis, we presented a time-dependent topological pattern in temporal variability of brain network in stroke patients with motor impairment. We also demonstrated the tight relationship between altered temporal variability of the ipsilesional precentral gyrus and motor recovery after stroke. Although the study was preliminary and in a modest sample size, our results support that measuring the temporal variability of brain network may be a potential tool for evaluating and predicting the motor recovery after stroke. These findings expand our understanding of the dynamic properties of brain networks and provide new insight into the underlying mechanisms of reorganization and integration of brain network over the recovery process after stroke.

## Figures and Tables

**Table 1:** Demographic and clinical data of stroke patients.

Patients (*n* = 19)	Acute stage	Subacute stage	Early chronic stage
Age (year)	52.26 ± 11.74 (30–71)	—	—
Sex (male)	17/19	—	—
Lesion side (left)	12/19	—	—
Lesion volume (ml)	3.26 ± 1.93 (0.61–6.44)	—	—
Days after stroke (day)	4.11 ± 1.76 (1–7)	10.47 ± 2.12 (8–14)	99.32 ± 8.54 (87–116)
UL-FMA	33.58 ± 14.55 (6–56)	42.63 ± 16.79 (7–61)	55.10 ± 12.27 (27–66)

UL-FMA: upper limb Fugl-Meyer assessment.

**Table 2:** Differences in regional temporal variability between controls and stroke patients at three stages.

Acute stage versus control			Subacute stage versus control			Early chronic stage versus control		
Brain region	*T* value	*P* value	Brain region	*T* value	*P* value	Brain region	*T* value	*P* value
*The ipsilesional side*								
PreCG	−3.556	<0.001	PoCG	−3.025	0.005	PreCG	−3.499	0.001
PoCG	−4.168	<0.001	ACG	−3.058	0.004	PoCG	−3.423	0.002
PCL	−3.915	<0.001	CRBL45	−3.158	0.003			
SMA	−4.056	<0.001						
SPG	−3.457	<0.001						
IPL	−3.572	<0.001						
ACG	−3.360	0.002						
MCG	−3.367	0.002						
Calcarine	−3.329	0.002						
Cuneus	−3.067	0.004						
ITG	−3.187	0.003						
Insula	−3.262	0.002						
FFG	−3.701	<0.001						
CRBL45	−3.852	<0.001						
*The contralesional side*								
PreCG	−3.309	0.002	SFGdor	−3.079	0.004	Hippocampus	−3.142	0.003
PoCG	−3.847	<0.001	Thalamus	−3.019	0.004			
SFGdor	−3.651	<0.001						
MFG	−3.211	0.003						
ACG	−3.276	0.002						
MCG	−3.201	0.003						
MOG	−3.442	<0.001						
MTG	−3.769	<0.001						
FFG	−3.369	0.002						
Thalamus	−3.347	0.002						
CRBL45	−3.775	<0.001						

ACG: anterior cingulate and paracingulate gyri; MCG: median cingulate and paracingulate gyri; ITG: inferior temporal gyrus; FFG: fusiform gyrus; MFG: middle frontal gyrus; SFGdor: superior frontal gyrus, dorsolateral; MOG: middle occipital gyrus; MTG: middle temporal gyrus; PreCG: precental gyrus; PoCG: postcentral gyrus; PCL: paracentral lobule; SPG: superior parietal gyrus; IPL: inferior parietal lobule; SMA: supplementary motor area; CRBL45: cerebelum_4_5.

**Table 3:** Differences in regional temporal variability among three stages.

Brain region	ANOVA		Multiple comparisons (post hoc Tukey's test)					
	*F* value		Subacute versus acute		Early chronic versus acute		Early chronic versus subacute	
	*P* value		Mean diff.	*P* value	Mean diff.	*P* value	Mean diff.	*P* value
*The ipsilesional side*								
PreCG	5.288	0.009	0.067	0.0154	—	—	−0.060	0.031
SMA	7.003	0.003	0.083	0.011	0.0815	0.013	—	—
SFGmed	6.377	0.004	0.064	0.013	0.063	0.015	—	—
Insula	5.436	0.009	0.073	0.010	0.059	0.042	—	—
FFG	6.458	0.004	0.074	0.005	0.059	0.026	—	—
SFGdor	6.575	0.004	—	—	0.081	0.003	—	—
*The contralesional side*								
MTG	10.829	<0.001	0.084	<0.001	0.046	0.041	—	—
STG	6.179	0.005	0.064	0.02	0.073	0.007	—	—
SMG	6.018	0.006	0.066	0.030	0.081	0.007	—	—
MOG	6.795	0.003	0.083	0.002	0.060	0.037	—	—
Cuneus	5.460	0.008	0.073	0.009	0.058	0.044	—	—
IFGoperc	5.965	0.006	0.062	0.034	—	—	—	—
Hippocampus	5.638	0.007	—	—	—	—	−0.053	0.007

Mean diff.: mean difference; PreCG: precental gyrus; SMA: supplementary motor area; SFGmed: superior frontal gyrus, medial; FFG: fusiform gyrus; SFGdor: superior frontal gyrus, dorsolateral; STG: superior temporal gyrus; SMG: supramarginal gyrus; MOG: middle occipital gyrus; IFGoperc: inferior frontal gyrus, opercular part.

## Supplementary Materials

Supplementary Materials Table 1: demographic and clinical data of stroke patients. Figure 1: the lesion locations of the stroke patients. The lesions are shown on axial slices of the diffusion-weighted images.
