# Supplementary material for: Dynamic Network Analysis Reveals Altered Temporal Variability in Brain Regions after Stroke: A Longitudinal Resting-State fMRI Study
Source: Neural Plast. 2018 Apr 5;2018:9394156. doi: 10.1155/2018/9394156 (PMC5907391; doi:10.1155/2018/9394156)
Supplement: Supplementary Materials — Table 1: demographic and clinical data of stroke patients. Figure 1: the lesion locations of the stroke patients. The lesions are shown on axial slices of the diffusion-weighted images. [file 9394156.f1.docx]

**Table 1**. Demograpic and clinical data of stroke patinents.

| Patient  ID | Gender | Age  (years) | Lesion  Side | Lesion  Location | Lesion  Volume  (ml) | Days after stroke | | | UL-FMA | | |
| --- | --- | --- | --- | --- | --- | --- | --- | --- | --- | --- | --- |
|  |  |  |  |  |  | **TP1** | **TP2** | **TP3** | **TP1** | **TP2** | **TP3** |
| 1 | M | 68 | R | CR | 4.99 | 5 | 11 | 97 | 33 | 56 | 66 |
| 2 | M | 50 | R | BG | 6.01 | 2 | 8 | 113 | 33 | 39 | 63 |
| 3 | M | 50 | R | BG | 3.54 | 7 | 12 | 103 | 56 | 61 | 64 |
| 4 | M | 49 | L | BG | 3.99 | 7 | 13 | 108 | 46 | 57 | 62 |
| 5 | M | 36 | L | CR | 3.26 | 6 | 13 | 105 | 26 | 43 | 62 |
| 6 | M | 43 | L | CR | 1.90 | 3 | 9 | 93 | 48 | 58 | 65 |
| 7 | M | 61 | L | CR | 5.80 | 6 | 12 | 112 | 40 | 43 | 55 |
| 8 | M | 39 | L | BG | 2.41 | 3 | 10 | 94 | 38 | 49 | 63 |
| 9 | F | 57 | R | IC | 0.75 | 1 | 8 | 94 | 35 | 47 | 63 |
| 10 | M | 65 | L | CR | 0.87 | 3 | 9 | 95 | 40 | 53 | 62 |
| 11 | M | 35 | R | CR | 4.56 | 3 | 8 | 116 | 6 | 7 | 35 |
| 12 | F | 64 | R | BG | 3.13 | 3 | 9 | 102 | 26 | 33 | 41 |
| 13 | M | 52 | L | CR | 4.57 | 4 | 9 | 105 | 12 | 12 | 38 |
| 14 | M | 30 | L | BG | 5.65 | 3 | 14 | 97 | 40 | 58 | 66 |
| 15 | M | 56 | L | CR | 1.54 | 3 | 14 | 91 | 48 | 51 | 58 |
| 16 | M | 48 | L | CR | 6.44 | 6 | 11 | 87 | 14 | 28 | 44 |
| 17 | M | 71 | L | BG | 1.96 | 4 | 9 | 92 | 36 | 39 | 48 |
| 18 | M | 61 | R | CR | 0.61 | 3 | 8 | 90 | 51 | 61 | 65 |
| 19 | M | 58 | L | IC | 1.21 | 6 | 12 | 93 | 10 | 15 | 27 |
| Mean(SE) |  | 52.26(11.74) |  |  | 3.26(1.93) | 4.11(1.76) | 10.47(2.12) | 99.32(8.54) | 33.58(14.55) | 42.63(16.79) | 55.10(12.27) |

CR, corona radiate;BG,basal ganglia; IC,internal capsule; SD,standard error. UL-FMA, upper limb Fugl-Meyer motor assessment.TP, time point.TP1, acute stage. TP2, Subacute stage. TP3, Early chronic stage.





**Fig.1.** The lesion locations of the stroke patients. The lesions are shown on axial slices of the diffusion-weighted images.
